# Pharmacotherapy of Adolescent Alcohol and Other Drug Use Disorders

**Published:** 1998

**Authors:** Ramon Solhkhah, Timothy E. Wilens

**Affiliations:** Ramon Solhkhah, M.D., is a fellow in child and adolescent psychiatry at Massachusetts General Hospital and McLean Hospital and a Clinical Fellow in Psychiatry at Harvard Medical School, Boston, Massachusetts. Timothy E. Wilens, M.D., is director of Substance Abuse Services in the Clinical Research Program in Pediatric Psychopharmacology at Massachusetts General Hospital and an associate professor of psychiatry at Harvard Medical School, Boston, Massachusetts

Medications are playing an increasingly important role as adjuncts to psychosocial strategies of alcoholism treatment. Although problems associated with the use of alcohol and other drugs (AODs) often appear in adolescence, most studies of AOD pharmacotherapy have been conducted in adults. Because youth may react differently from adults to both the therapeutic and potentially harmful effects of medications, the applicability of existing data to youth who abuse AODs remains unclear. This article explores strategies for using medications to treat AOD use disorders^1^ in youth, focusing on alcoholism when relevant data are available.

A systematic search of published research revealed 10 studies on the effects of medication for treating children or adolescents with AOD disorders. The studies differ significantly in experimental methods and in the definition and measurement of treatment outcome. Two were controlled studies, a type of study that compares the effects of the experimental medication with those of a sham medication, or placebo. Four were open trials, which evaluate experimental medication without a placebo control. Four were case reports, which describe the responses of one or a few patients to specific treatments. Treatment outcomes were evaluated using patient questionnaires; random urine screens to detect AOD use; and overall ratings of improvement in the patient’s addictive behavior, co-occurring psychiatric symptoms, and psychosocial functioning. Although uncontrolled studies and case reports may suggest directions for research, controlled studies are needed to evaluate the effects of a medication.

Pharmacotherapeutic strategies for treating AOD disorders, as described in this sidebar, include craving reduction, substitution therapy, aversive therapy, and treatment of underlying psychiatric conditions (Kaminer 1995). Because subjects in all the studies discussed received concurrent nonpharmacological treatment, this article describes effects presumably attributable to the medications themselves.

## Craving Reduction

Many researchers consider craving a fundamental aspect of addiction. Various definitions of craving exist based on behavioral, neurological, and subjective psychological criteria. In this article, the term “craving” refers to the urge or desire for a particular AOD. Craving can be evaluated experimentally by behavioral responses in laboratory animals or by self-reported mental states in human subjects. The reduction of craving through pharmacotherapy may help prevent relapse in patients undergoing treatment for alcoholism or other addictions.

Among the best known medications for treating alcoholism is naltrexone (ReVia^TM^ or Trexan^®^). Naltrexone blocks certain communication pathways in the brain that are involved in the development of addiction to both alcohol and abused opiates (e.g., heroin). In studies of alcohol-dependent adults, naltrexone decreased alcohol consumption by approximately one-half (Volpicelli et al. 1997). The only study on the use of naltrexone in AOD-abusing youth is a case report of a 17-year-old alcohol-dependent male who remained abstinent during a brief (30-day) trial of naltrexone (Wold and Kaminer 1997). Nalmefene (a naltrexone derivative) and acamprosate also show promise for reducing alcohol craving in adults (Gastfriend et al. 1998). The efficacy of those medications in adolescents has not been determined.

Additional medications that may modify AOD craving include serotonin reuptake inhibitors (SRIs) and tricyclic antidepressants (e.g., desipramine [Norpramin^®^]), which are used to treat some psychiatric conditions in both adult and juvenile patients (Wilens et al. 1998). Studies of SRIs to reduce craving for cocaine, opiates, and alcohol in adults yielded mixed results (Gastfriend et al. 1998). In case reports, desipramine administration led to overall functional improvement in cocaine-dependent adolescents (Kaminer 1994), possibly by treating patients’ underlying psychiatric disorders (see the section entitled “Treatment of Underlying Psychopathology”).

## Substitution Therapy

In substitution therapy, the patient’s drug of abuse is replaced with the supervised administration of a related medication. Substitution therapy is predicated on the assumption that replacing AODs with prescribed medications will shift control of AOD use to the patient and clinician while reducing the patient’s need to maintain behaviors associated with addiction (e.g., illicitly obtaining drugs). An established treatment for opiate dependence in adults is its replacement by methadone, a synthetic opiate that unlike heroin, causes minimal euphoria, drowsiness, or depression. Unfortunately, no systematic data exist on opiate substitution in adolescents, and Federal regulations severely restrict the use of methadone in youth (Gastfriend et al. 1998).

Research suggests that cocaine dependence may possibly be treated by substitution therapy using psychostimulants. Those medications differ from cocaine in chemical structure but have some behavioral and emotional effects in common with it. The psychostimulant methylphenidate (Ritalin^®^) either led to no change or decreased cocaine use in adults with and without co-occurring psychiatric disorders (specifically attention deficit/hyperactivity disorder) (Grabowski et al. 1997; Levin et al. 1997) (see article by Wilens, pp. 127–130). The extrapolation of those findings to cocaine-abusing youth remains unstudied.

No substitution therapies exist for alcohol. However, the use of medications during alcohol detoxification is predicated on a similar concept. The brain adapts to heavy or prolonged alcohol consumption by developing tolerance to some of alcohol’s effects. The mechanisms of tolerance to alcohol-induced sedation may contribute to the symptoms of nervous system hyperactivity that occur when alcohol consumption suddenly ceases (i.e., withdrawal).^2^ Diazepam (Valium^®^) and related sedative medications (i.e., benzodiazepines) alleviate withdrawal symptoms in adults. Therefore, despite a lack of systematic experimental data, adolescents manifesting alcohol withdrawal symptoms are often treated with benzodiazepines.

## Aversive Therapy

The goal of aversive therapy is to reduce AOD use through medications that lead to unpleasant responses following the consumption of the abused drug. In the United States, aversive treatment for alcoholism generally involves disulfiram (Antabuse^®^). This medication interferes with the body’s metabolism of alcohol, resulting in the accumulation of a toxic by-product called acetaldehyde. Alcohol consumption in the presence of disulfiram results in flushing, nausea, vomiting, headache, and chest discomfort; less commonly, it results in chest pain, palpitations, difficulty breathing, dizziness, confusion, and low blood pressure (Gastfriend et al. 1998; Myers et al. 1994). Rare serious reactions can include irregular heartbeat, heart failure, respiratory depression, seizures, and death. Alcohol consumption may produce those effects for up to 2 weeks after the patient has stopped taking disulfiram.

In a case study, Myers and colleagues (1994) administered disulfiram (250 milligrams daily) to two alcohol-dependent adolescents. One subject remained abstinent for 4 months before discontinuing disulfiram and relapsing to alcoholism. The other subject failed to take the medication and continued drinking. The problem of noncompliance with treatment suggests that disulfiram may be most useful in residential (i.e., inpatient) treatment facilities (e.g., for use on weekend passes). Disulfiram should be administered, under supervision, only to patients who are medically healthy, intellectually competent, insightful about their AOD problem, and highly motivated to achieve recovery.

## Treatment of Underlying Psychopathology

Evidence suggests that some people may use AODs in part to self-medicate distressing mental states associated with underlying psychiatric conditions (Khantzian 1997). Adolescents with AOD disorders exhibit a high prevalence of psychiatric disorders compared with the general population (see article by Clark and Bukstein, pp. 117–126). Disorders that co-occur with AOD abuse include depressive and anxiety disorders, bipolar disorder, attention deficit/hyperactivity disorder (ADHD) (see article by Wilens, pp. 127–130) and conduct disorder^3^ (Bukstein et al. 1989; Weinberg et al. 1998). Few studies exist on the pharmacological treatment of those conditions in AOD-abusing youth.

Findings from open trials suggest that the psychostimulant pemoline (Cylert^®^) and the antidepressant bupropion (Wellbutrin^®^ and others) may ameliorate symptoms of ADHD in AOD-abusing youth with co-occurring conduct disorder. Because those studies were completed in inpatient settings, however, effects on AOD use were not determined (Leon et al. 1998; Riggs et al. 1996). Methylphenidate improved both AOD disorders and co-occurring ADHD in adolescents and young adults (Grabowski et al. 1997; Levin et al. 1997; Riggs 1998).

Mood disorders in youth are among the most difficult of psychiatric disorders to treat. In a small open trial (Riggs et al. 1997), the SRI fluoxetine (Prozac^®^) reduced depressive symptoms in adolescents with AOD disorders while producing no significant adverse side effects. Bipolar disorder is characterized by marked mood swings that range from severe depression to a state of abnormal mental excitement and physical energy (i.e., mania). In adolescents, mania is generally manifested by aggressivity and behavioral outbursts. Medications for bipolar disorder include lithium; certain anticonvulsants, such as carbamazapine (Tegretol^®^), valproic acid (Depakote^®^), and gabapentin (Neurontin^®^); and antipsychotic medications, such as thioridazine (Mellaril^®^) and olanzapine (Zyprexa^®^) (Wilens et al. 1998).

Patterns of AOD abuse among bipolar youth suggest an attempt to self-medicate manic symptoms. Results of recent research are consistent with this possibility. In controlled trials, Geller and associates (1998) administered lithium to adolescents with comorbid bipolar and AOD use disorders. After 6 weeks of treatment, lithium-treated subjects exhibited significant reductions in AOD use compared with subjects administered a placebo. Decreased AOD use correlated with improvements in overall functioning consistent with increased stability of mood. Similarly, adolescents subject to temper outbursts and irritability reduced their marijuana consumption after their moods were stabilized with valproic acid (Donovan et al. 1996).

## Safety and Monitoring

Appropriate pharmacotherapy for adolescent AOD use disorders depends on the medication’s effectiveness, potential for abuse, possible interactions with other medications or AODs, and factors that affect patient compliance (e.g., unpleasant side effects). The risks of pharmacotherapy are highlighted by a recent report of an adverse drug interaction between marijuana and tricyclic antidepressants (Wilens et al. 1997). In addition, a systematic assessment of a nonresidential child psychiatry clinic revealed that 25 percent of adolescent AOD-abusing patients were entirely noncompliant with pharmacotherapy and 8 percent had abused their prescribed medication or had made it available to others for illicit use (Wilens et al. 1996). Clinicians should therefore monitor patients’ progress by objective measures whenever possible. For example, blood tests may be used to confirm whether the patient is taking prescribed doses of medications, and frequent random urine screens may detect illicit AOD use.

Research results, coupled with clinical experience, suggest the importance of tailoring the choice of medication and the course of treatment and followup to the needs of the individual patient. Coordination of pharmacotherapy with family members and other caregivers can enhance treatment outcome. Additional controlled studies are essential to assess the effectiveness and safety of pharmacotherapy both alone and in combination with other addiction interventions.

## Footnotes

1 AOD disorders considered here include addiction (i.e., dependence) and abuse.

2 Withdrawal symptoms include anxiety, tremors, increased heart rate and, in some cases, convulsions.

3 For definitions of psychiatric syndromes, see article by Clark and Bukstein, pp. 117–126.
